# Temporal patterns of total, animal and plant protein intakes of Australian adults: a latent class analysis

**DOI:** 10.1007/s00394-026-03918-8

**Published:** 2026-02-12

**Authors:** Hesti Retno Budi Arini, Sarah A. McNaughton, Sze-Yen Tan, Rebecca M. Leech

**Affiliations:** 1 Present Address: Institute for Physical Activity and Nutrition (IPAN), School of Exercise and Nutrition Sciences, Deakin University, Geelong, VIC 3220 Australia; 2 School of Exercise and Nutrition Sciences, Deakin University, Geelong, VIC 3220 Australia; 3 Health and Well-Being Centre for Research Innovation, School of Human Movement and Nutrition Sciences, University of Queensland, St Lucia, QLD 4067 Australia

**Keywords:** Dietary protein, Eating patterns, Eating occasion, Latent class analysis

## Abstract

**Purpose:**

Few studies have investigated temporal protein patterns of different protein sources. This study aimed to describe temporal patterns of total, plant, and animal protein intake of Australian adults and examine their associations with sociodemographic and eating pattern characteristics.

**Methods:**

Total, animal, and plant protein intakes were estimated from the Australian National Nutrition and Physical Activity Survey 2011–12 dietary recall data (≥ 19 years; n = 6741). Separate latent class models were used to determine the patterns based on hourly intakes of total, animal, and plant protein. Pearson’s Chi-square test and one-way analysis of variance were used to examine the differences in characteristics between latent classes of the patterns.

**Results:**

Three sex-stratified temporal patterns of total (T1-T3), animal (A1-A3), and plant (P1-P3) proteins were identified. Class 1 was characterised by higher probabilities of consuming protein at the typical Australian mealtime (e.g., at 18:00 h for A1), while Class 2 had higher probabilities of intake 1-h later (e.g., 19:00 h for A2). Class 3 was characterised by variable timing (e.g., 17:00 h or 20:00 h for A3). Adults in Class T1, A1, and P1 were older than other classes, whereas those in Class T2 and A2 had higher incomes (all *p* < 0.001). Adults in Class T3, A3, and P3 were younger and had lower total, animal, and plant protein intakes from meals (all *p* < 0.001) but higher intakes from snacks (*p* < 0.001), except for women’s animal protein.

**Conclusion:**

This study identified three temporal protein patterns, which varied by age, income, meal frequency, and protein intake at meals and snacks. Future research needs to examine whether these patterns have different health implications.

**Supplementary Information:**

The online version contains supplementary material available at 10.1007/s00394-026-03918-8.

## Introduction

Timing of food and nutrient intake has been an emerging research focus, mainly due to its potential influence on diet and health [1, 2]. For instance, having lunch later in the day has been shown to be associated with poor weight regulation and insulin sensitivity [2]. When hourly energy intake was assessed, adults with later but more frequent eating occasions (EOs) across the day—defined as the grazing pattern—had lower diet quality than those following the conventional pattern (e.g., lunch at noon and dinner at 6 PM) [1]. Temporal eating patterns, which include timing, frequency, and distribution of eating occasions across the day [3], may also be considered important given the interplay between dietary intake, appetite, and metabolism with circadian rhythm [4].

In addition to the distribution of energy intake across the day, previous evidence also suggested the influence of temporal protein patterns on health. Most observational studies focused on protein distribution among older adults, suggesting the need for higher protein intake at breakfast or lunch for optimal muscle protein synthesis [5, 6]. Given the different anabolic effects between animal and plant protein, examining temporal patterns of source-specific protein might help improve protein distribution across the day for better muscle protein synthesis by considering both the amount and sources of protein [6]. While Hengeveld et al. [5] reported dairy, cereal, and meats being the main protein sources at EOs of older adults with both low (< 0.8 g/kg body weight/d) and high (≥ 0.8 g/kg body weight/d) total daily protein intake, the temporal distribution of protein sources has not been explored yet.

With respect to cardiometabolic-related outcomes, one cross-sectional study reported an inverse association between adults’ protein intake at breakfast with blood pressure, and a positive association between protein intake at dinner with insulin sensitivity [7], while another cross-sectional study reported a positive association between protein intake at night and obesity [8]. While the associations with cardiometabolic outcomes might also differ by dietary patterns containing different combinations of foods, evidence from feeding studies [9] on macronutrient order suggests protein intake timing may be important. However, none of the associations in previous studies of temporal protein patterns [7, 8] considered the timing of protein intake from different food sources, and therefore, the source-specific temporal protein patterns warrant future studies given the differential relationships of animal v. plant protein with cardiometabolic health [7].

From the methodological perspective, most previous studies on temporal protein patterns relied on self-reported EOs, where EOs were self-defined as breakfast, lunch, dinner, and snacks but with limited use of specified eating times, which might not always represent the actual timing of intake at these EOs between participants [5–7, 10]. While self-defined EOs may be more routinely collected in dietary studies, this approach could be influenced by participants’ subjectivity in classifying their eating events as certain meals or snacks due to their different cultures and work/sleep schedules [3, 4]. Furthermore, knowing specific eating hours could provide additional information on temporal protein distribution across 24 h as participants may use the same term for the eating event, yet this might vary in actual timing and in relation to their other meals. Combining self-reported EOs and specific eating timing information may help our understanding of the patterns of protein intake in the population and its potential association with health. For example, a study examining protein intake at self-defined meals and snacks reported non-significant associations with BMI [7], but another study that utilised timing of intake found protein consumed at dinner after 8 PM was associated with higher BMI [11].

Data-driven methods such as latent class analysis (LCA) have recently been used to identify eating patterns. Data-driven methods allow the data to identify patterns by summarising many variables without pre-selecting cut-points [12], and therefore involves less reliance on pre-defined ideas or previous knowledge [13]. Specifically, LCA describes the probabilities of observed variables being varied across groups of individuals where patterns of that group membership are unknown [12]. It has previously been used in nutrition to identify food-based dietary patterns in Brazilian adults [14], and temporal eating patterns of Australian and Iranian adults.[15, 16] Therefore, this method may be useful for describing the temporal distribution of protein intake at EOs by using hourly protein intake of individuals across the day.

Temporal protein patterns have been shown to vary between and within countries, and this may be related to cultural and sociodemographic influences on dietary behaviours. For example, protein intake of American adults was highest at dinner [7], while Mexican adults had their highest protein intake at lunch [6], which is possibly due to differences in timing of consumption of the main (i.e., largest) meal [17, 18]. Further, in Finnish adults, protein intake timing of the morning meal varied by working and non-working days [19]. Therefore, this study aimed to describe patterns of total, plant, and animal protein intake at EOs in Australian adults using the LCA, and to examine these patterns according to their sociodemographic and eating pattern characteristics (e.g., frequencies and amount of hourly protein intake).

## Methods

### Sample and study design

This secondary data analysis used data from the Australian National Nutrition and Physical Activity Survey (NNPAS) 2011–12, which was conducted across eight states and territories by the Australian Bureau of Statistics (ABS) [20]. The survey design was a stratified multistage area of private dwellings with probability sampling design [20]. The NNPAS included 12,153 participants, but this study only focused on adults aged ≥ 19 years (*n* = 9341). Shift workers were excluded to account for the potentially unique eating patterns in this subgroup that differ from the general population. Nonetheless, the small proportion of shift workers in this study population did not allow separate examination of their patterns. Participants with no dietary data, and pregnant or lactating women were also excluded, which resulted in 6741 adults being analysed in this study.

### Ethics statement

This study was conducted according to the guidelines in the Declaration of Helsinki. The ethics approval for the ABS was provided through the Census and Statistics Act 1905 in conducting the survey, including the interview component of the NNPAS [20]. Informed consent was sought from all individual participants by completing a consent form [20]. Deidentified data was used in all secondary data analyses in this study, and an exemption from ethics review was granted by the Deakin University Human Research Ethics Committee (DUHREC no. 2023-135).

### Dietary assessment

The dietary data of NNPAS was collected through Computer Assisted Personal Interview (CAPI) for the first 24-h recalls by trained interviewers adopting the USDA Automated Multiple 5-Pass Method (AMPM) [20]. The sample in this dietary recall was spread across a 12-month enumeration period to consider possible seasonal effects, with 19% of interviews being conducted in spring, 23% in winter, 27% in summer, and 31% in autumn [20]. The day distribution of recalls was 14–18% on weekdays, 11.5% on Saturdays, and 3.5% on Sundays [20]. For each 24-h recall, participants were requested to report their food and beverage intakes, as well as eating occasions, amount, and time of consumption [20]. The second 24-h recall was conducted during a telephone interview at least eight days after the first 24-h recall [20]. Considering the response rate (63%) of the second 24 h-recall and the likelihood of sample bias, only dietary data from adults completing the first 24-h recall day was used in this analysis to preserve the nationally representative sample.

### Total, plant and animal protein intake

Total protein intake was obtained from nutrient intake calculations on the first recall day referring to the AUSNUT13 food nutrient database [21]. In terms of plant v. animal protein classification, foods from the 2011–13 Australian Health Survey Food and Supplement Classification (n = 5740) were classified as plant or animal protein sources by referring to the Food Standards Australia New Zealand (FSANZ) major and sub-major groups codes [21]. Two approaches were used to define whether certain food items are considered plant or animal protein sources, i.e., 1) Plant-based protein consisting of grains, nuts, vegetables and other plant-based, protein-containing foods, and 2) All animal-source foods, including meats, fish, and dairy products. Plant and animal protein in mixed dishes were estimated using AUSNUT 2011–13 food recipe file, food details file, and Australian Dietary Guidelines (ADG) food classification system, which involved initial classification by one researcher and agreement with the other three researchers upon the discussions [22, 23]. All protein intake was expressed in grams/day (g/d).

### Temporal protein pattern

Temporal patterns of total, animal, and plant protein were identified across eating occasions, where an eating occasion (EO) was defined as an occasion with any food and beverage consumption containing ≥ 210 kJ and separated by 15 min from the preceding and succeeding EO [24]. Following this, the protein intake of the included EOs (i.e., ≥ 210 kJ) was estimated. Using this information on protein intake, binary variables were created indicating whether participants had protein intake at each hour of the day (i.e., “no” if the EO had zero grams of protein, otherwise “yes”), separately for total, animal, and plant protein. Separate continuous variables for total, animal, and plant protein were also created using the amount (g) of hourly intake among participants who consumed protein. All protein continuous variables were winsorised at a 2% level to address outliers, resulting in binary and continuous variables of protein intake from 5 AM to midnight (for total and plant protein) and from 5 AM to 11 PM (for animal protein) being used in the latent class identification.

### Sociodemographic characteristics

Given the different protein intake across age, ethnicity, socio-economic status, and household structure shown in the previous studies [25–27], the information on participants’ age (in years), country of birth, geographic region, Socio-economic Indexes for Areas (SEIFA), income, employment, education level, marital status, and household composition obtained from the household survey [20] was used in this study. The ABS classified country of birth as (1) Australia, (2) other main English-speaking countries, and (3) all other countries, while geographic regions included (1) major cities, (2) inner regional, and (3) other regional of Australia which included outer regional, remote, and very remote Australia [20]. SEIFA ranked Australia’s areas according to relative socio-economic advantage and disadvantage, with lower quintiles indicating greater disadvantage [28]. The income categorical variable was obtained by collapsing the Australian weekly income deciles provided in the household survey [20]. Employment categories included adults who were (1) employed, (2) unemployed, and (3) not in the labour force [20]. Education level was classified as (1) low, for adults with no non-school qualification, (2) medium, for adults who completed high school and/or certificate/diploma, and (3) high, for adults with a tertiary qualification [15, 20]. Marital status included (1) married adults, either in a registered or de facto marriage, and (2) not married adults, and household composition included (1) Person living alone; (2) Couple only; (3) Couple family with children; and (4) All other households (merged with “One parent family with children” and “Unrelated persons aged 15+ only” types due to the small cell counts) [20].

### Eating pattern characteristics

EO self-reported by participants as meals included breakfast, lunch, dinner, brunch, and supper, while snacks included snack, morning/afternoon tea, and any beverage/break occasion [24]. The mean frequencies of total EOs, meals, and snacks were calculated separately for total, animal, and plant protein. Overall protein intake and protein intake at meals and snacks were also calculated for total, animal, and plant protein, expressed in grams and proportions of energy intake.

### Statistical analysis

#### Latent classes of temporal protein patterns

The two-part latent variable mixture modelling (LVMM) approach, an extension of LCA, was performed in M-Plus v.8.7 to identify latent classes of temporal protein patterns. The classes were identified separately for men and women accounting for the different energy and protein requirements between sexes [29]. The two-part option was used because of the semi-continuous distribution of hourly protein intake, with a high number of zero values at certain hours [30]. Both binary and continuous variables were used as the separate input variables for total, animal, and plant protein, and square-root transformation was applied to all models to improve the normal distribution of hourly protein intake. Only the person weights of NNPAS were included in the LVMM models because a replicate weights option is not currently available for the mixture analysis in MPlus. Models with 2 latent classes were tested, and additional classes were added until the optimal number of latent classes was reached. A range of model fit indices, including (1) Sample-size adjusted Bayesian Information Criterion, where smaller values suggest better models; (2) Entropy, where value > 0.80 suggests better separation between classes; (3) latent class posterior probabilities, where probability > 0.80 suggests good classification accuracy; and (4) class interpretability were used to determine the optimal number of latent classes [31].

#### Associations between temporal protein patterns, eating pattern and sociodemographic characteristics

Analyses for the associations of temporal protein patterns with eating pattern and sociodemographic characteristics were conducted using Stata v.18. Person weights and replicate weights were applied to the descriptive analyses, and descriptive statistics for sample characteristics were presented as weighted means or weighted percentages. Pearson’s Chi-square test (for categorical variables) and one-way analysis of variance (for continuous variables) with Bonferroni correction were used to examine the pairwise differences in participant characteristics between latent classes of temporal protein patterns. Differences were considered significant at *p* < 0.05.

## Results

A three-class model for men’s and women’s total (Classes T1-T3), animal (Classes A1-A3), and plant protein (Classes P1-P3) intakes was selected to describe temporal protein patterns by considering model fit indices, as shown in Table 1. Adjusted BIC values were lower for the 3-class models, and the entropy values (≥ 0.8) and class probabilities (≥ 0.8) also supported a 3-class model.

**Table 1:** Model fit indices for latent class models of temporal protein patterns

	Men		Women	
	2 classes	3 classes	2 classes	3 classes
Total protein				
Loglikelihood	− 57579.984	− 57137.578	− 63158.753	− 62708.004
Entropy	0.931	0.838	0.925	0.898
SSABIC*	115628.807	114934.463	126797.832	126091.465
Latent class probabilities	Class 1 = 0.994 Class 2 = 0.974	Class 1 = 0.946 Class 2 = 0.954 Class 3 = 0.892	Class 1 = 0.968 Class 2 = 0.994	Class 1 = 0.955 Class 2 = 0.934 Class 3 = 0.977
Animal protein				
Loglikelihood	− 40057.786	− 39627.075	− 43641.936	− 43258.107
Entropy	0.860	0.856	0.868	0.851
SSABIC	80559.992	79879.269	87739.181	87156.647
Latent class probabilities	Class 1 = 0.905 Class 2 = 0.986	Class 1 = 0.989 Class 2 = 0.970 Class 3 = 0.902	Class 1 = 0.938 Class 2 = 0.989	Class 1 = 0.953 Class 2 = 0.978 Class 3 = 0.920
Plant protein				
Loglikelihood	− 44547.760	− 44157.643	− 49065.321	− 48695.008
Entropy	0.880	0.793	0.956	0.797
SSABIC	89564.360	88974.592	98610.967	98065.473
Latent class probabilities	Class 1 = 0.958 Class 2 = 0.987	Class 1 = 0.946 Class 2 = 0.875 Class 3 = 0.918	Class 1 = 0.997 Class 2 = 0.980	Class 1 = 0.945 Class 2 = 0.905 Class 3 = 0.913

^*^SSABIC sample size adjusted Bayesian Information Criterion

###  Latent classes of temporal total protein patterns

**Fig. 1:**
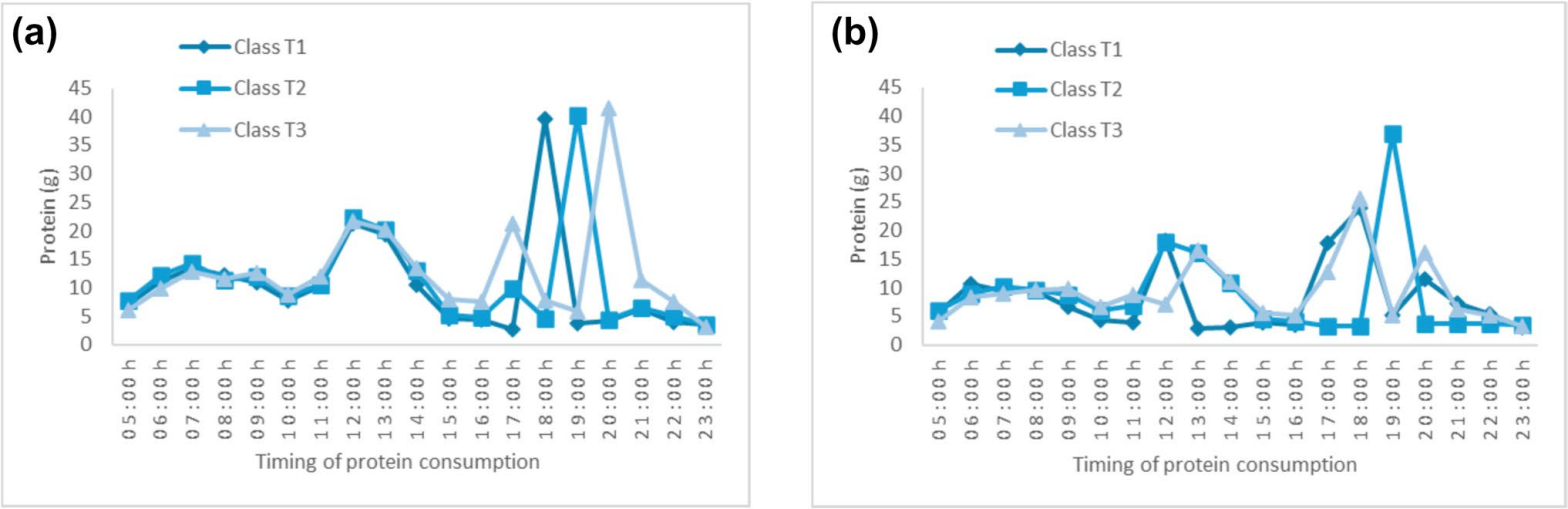
Total protein intake of men** a** and women **b**

Temporal total protein patterns were distinguished by conditional probabilities for consuming protein at evening hours (Supplementary Material 1 and 2). Despite variations in conditional probabilities and intake amount, all three patterns had higher total protein intake in the evening (Supplementary Material 1: Fig. 1a, b and Fig. 1a, b), and therefore no class labels were specified. Men’s T1 pattern was characterised by higher probabilities of consuming total protein at hours when Australians commonly have evening meals (18:00–19:00 h), while men’s T2 pattern had higher probabilities of eating total protein an hour later than the T1 pattern’s evening mealtime (19:00–20:00 h). Men’s T3 pattern consumed total protein either before (17:00–18:00 h) or after (20:00–21:00 h) the evening mealtime of the other patterns, but their probabilities of consuming protein were lower (< 0.5). Similarly, women’s T1 pattern was more likely to eat total protein at typical evening mealtime and women’s T2 pattern had higher probabilities of consuming total protein 1-h later. Women’s T3 pattern had total protein at the same evening mealtime of T1 or an hour later than the time of T2, with lower conditional probabilities.

Total protein patterns of both sexes were associated with different sociodemographic and eating pattern characteristics (Table 2). T1 patterns of both sexes were more likely to be older (*p* < 0.001), and most of them were married (*p* < 0.001, men only). A higher proportion of men and women with T2 pattern had higher socioeconomic status (Highest SEIFA quintile: men *p* = 0.006, women *p* < 0.001), income and employment levels (*p* < 0.001) compared to those with T1 and T3 patterns. Compared to the T1 pattern, men and women with the T3 pattern were younger and had lower proportions of married adults (men only, *p* < 0.001). T3 patterns of both sexes were also associated with lower EO frequency, meal frequency, and protein intake from meals (*p* < 0.001), but higher intake from snacks (men *p* < 0.001, women *p* = 0.03).

**Table 2:** Sociodemographic characteristics of Australian men and women, by total protein latent class membership

Characteristic	Men (N = 3169)				Women (N = 3572)			
	Class T1	Class T2	Class T3	*p*-value	Class T1	Class T2	Class T3	*p*-value
n(%)	1086 (34.3%)	1140 (35.9%)	943 (29.8%)		1102 (30.9%)	1042 (29.2%)	1428 (39.9%)	
Age [year (mean, SD)]*	52.2 (17.4)^a^	48.1 (16.3)^b^	46.6 (17.7)^b^	< 0.001	53.0 (18.4)^a^	47.8 (16.3)^b^	49.4 (17.4)^b^	< 0.001
Country of Birth [n(%)]^a^				0.011				0.001
Australia	794 (73.1%)	785 (68.9%)	631 (66.9%)		809 (73.5%)	780 (74.9%)	977 (68.4%)	
Main English-Speaking Countries (Canada, Ireland, NZ, South Africa, UK, USA)	141 (13.0%)	161 (14.1%)	128 (13.6%)		125 (11.3%)	124 (11.9%)	178 (12.5%)	
Other	151 (13.9%)	194 (17.0%)	184 (19.5%)		168 (15.2%)	138 (13.2%)	273 (19.1%)	
Geographic region				< 0.001				0.19
Major cities of Australia	699 (64.4%)	750 (65.8%)	651 (69.0%)		669 (60.7%)	676 (64.9%)	919 (64.4%)	
Inner regional Australia	241 (22.2%)	189 (16.6%)	135 (14.3%)		244 (22.1%)	199 (19.1%)	296 (20.7%)	
Other	146 (13.4%)	201 (17.6%)	157 (16.7%)		189 (17.2%)	167 (16.0%)	213 (14.9%)	
SEIFA [n(%)]				0.006				< 0.001
Lowest 20%	207 (19.1%)	175 (15.4%)	191 (20.3%)		232 (21.1%)	155 (14.9%)	300 (21.0%)	
Second quintile	216 (19.9%)	221 (19.4%)	197 (20.9%)		257 (23.3%)	205 (19.7%)	265 (18.6%)	
Third quintile	226 (20.8%)	213 (18.7%)	190 (20.1%)		214 (19.4%)	198 (19.0%)	303 (21.2%)	
Fourth quintile	198 (18.2%)	213 (18.7%)	160 (17.0%)		172 (15.6%)	200 (19.2%)	241 (16.9%)	
Highest 20%	239 (22.0%)	318 (27.8%)	205 (21.7%)		227 (20.6%)	284 (27.2%)	319 (22.3%)	
Income^b^				< 0.001				< 0.001
First quintile	207 (20.4%)	161 (15.1%)	171 (19.5%)		284 (28.7%)	163 (16.9%)	356 (27.2%)	
Second quintile	208 (20.5%)	133 (12.4%)	145 (16.6%)		231 (23.3%)	169 (17.5%)	264 (20.2%)	
Third quintile	210 (20.6%)	184 (17.2%)	127 (14.5%)		154 (15.6%)	166 (17.2%)	239 (18.3%)	
Fourth quintile	222 (21.9%)	259 (24.2%)	192 (21.9%)		180 (18.2%)	226 (23.4%)	226 (17.3%)	
Fifth quintile	168 (16.6%)	332 (31.1%)	241 (27.5%)		141 (14.2%)	243 (25.0%)	223 (17.0%)	
Employment				< 0.001				< 0.001
Employed	672 (61.8%)	850 (74.6%)	648 (68.7%)		507 (46.0%)	694 (66.6%)	765 (53.6%)	
Unemployed	31 (2.9%)	22 (1.9%)	28 (3.0%)		18 (1.6%)	22 (2.1%)	46 (3.2%)	
Not in the labour force	383 (35.3%)	268 (23.5%)	267 (28.3%)		577 (52.4%)	326 (31.3%)	617 (43.2%)	
Education level				< 0.001				< 0.001
Low	234 (21.5%)	308 (27.0%)	258 (27.4%)		264 (24.0%)	344 (33.0%)	362 (25.4%)	
Medium	469 (43.2%)	433 (38.0%)	322 (34.1%)		271 (24.5%)	314 (30.1%)	438 (30.6%)	
High	383 (35.3%)	399 (35.0%)	363 (38.5%)		567 (51.5%)	384 (36.9%)	628 (44.0%)	
Marital status				< 0.001				0.08
Married^§^	685 (63.1%)	691 (60.6%)	458 (48.6%)		574 (52.1%)	537 (51.5%)	686 (48.0%)	
Not married	401 (36.9%)	449 (39.4%)	485 (51.4%)		528 (47.9%)	505 (48.5%)	742 (52.0%)	
Household composition				< 0.001				0.19
Person living alone	256 (23.6%)	258 (22.6%)	296 (31.4%)		311 (28.2%)	279 (26.8%)	388 (27.2%)	
Couple only	390 (35.9%)	374 (32.8%)	246 (26.1%)		324 (29.4%)	269 (25.8%)	376 (26.3%)	
Couple family with children	320 (29.5%)	359 (31.5%)	256 (27.1%)		260 (23.6%)	288 (27.6%)	363 (25.4%)	
All other households	120 (11.0%)	149 (13.1%)	145 (15.4%)		207 (18.8%)	206 (19.8%)	301 (21.1%)	
EO frequency	4.6 (0.9)^a^	4.6 (0.9)^a^	4.3 (1.0)^b^	< 0.001	4.7 (0.8)^a^	4.7 (0.9)^a^	4.4 (0.9)^b^	< 0.001
Meal frequency	3.2 (0.8)^a^	3.1 (0.8)^b^	2.9 (0.9)^c^	< 0.001	3.3 (0.7)^a^	3.2 (0.8)^a^	3.0 (0.8)^b^	< 0.001
Snack frequency	1.4 (0.5)	1.5 (0.5)	1.4 (0.5)	0.75	1.4 (0.5)^a^	1.5 (0.5)^b^	1.5 (0.5)^a^	0.002
Total protein intake (g)	103.2 (47.0)	104.1 (48.1)	100.1 (45.7)	0.15	78.5 (33.6)^a^	86.3 (33.1)^b^	74.3 (35.4)^c^	< 0.001
Total protein intake (%EI)	18.0 (5.4)	17.8 (6.0)	17.6 (6.2)	0.23	18.2 (6.0)	18.6 (6.1)^a^	17.7 (6.3)^b^	0.001
Total protein intake from meals (g)	90.0 (41.7)^a^	89.8 (42.0)^a^	83.3 (41.0)^b^	< 0.001	68.3 (32.3)^a^	75.8 (31.5)^b^	63.3 (33.1)^c^	< 0.001
Total protein intake from meals (%EI)	15.9 (5.6)^a^	15.5 (6.1)^a^	14.9 (6.5)^b^	< 0.001	15.9 (6.3)^a^	16.5 (6.3)^a^	15.2 (6.5)^b^	< 0.001
Total protein intake from snacks (g)	13.5 (16.4)^a^	14.7 (21.3)^a^	17.8 (22.7)^b^	< 0.001	10.4 (10.0)^a^	10.9 (10.9)	11.7 (13.5)^b^	0.03
Total protein intake from snacks (%EI)	2.1 (2.2)^a^	2.3 (2.6)^a^	2.9 (3.3)^b^	< 0.001	2.3 (2.2)^a^	2.2 (2.0)^a^	2.7 (2.9)^b^	< 0.001

^*^Differences between classes for continuous variables were assessed by using analysis of variance with Bonferroni correction, and different superscript letters indicate significant differences between classes. Values are weighted means (SDs)

^a^Differences between classes for categorical variables were assessed by using adjusted Pearson’s chi-square test. Values are weighted percentages

^b^n = 2960 men and n = 3265 women due to missing cases for income

^§^A registered or de facto marriage

### Latent classes of temporal animal protein patterns

**Fig. 2:**
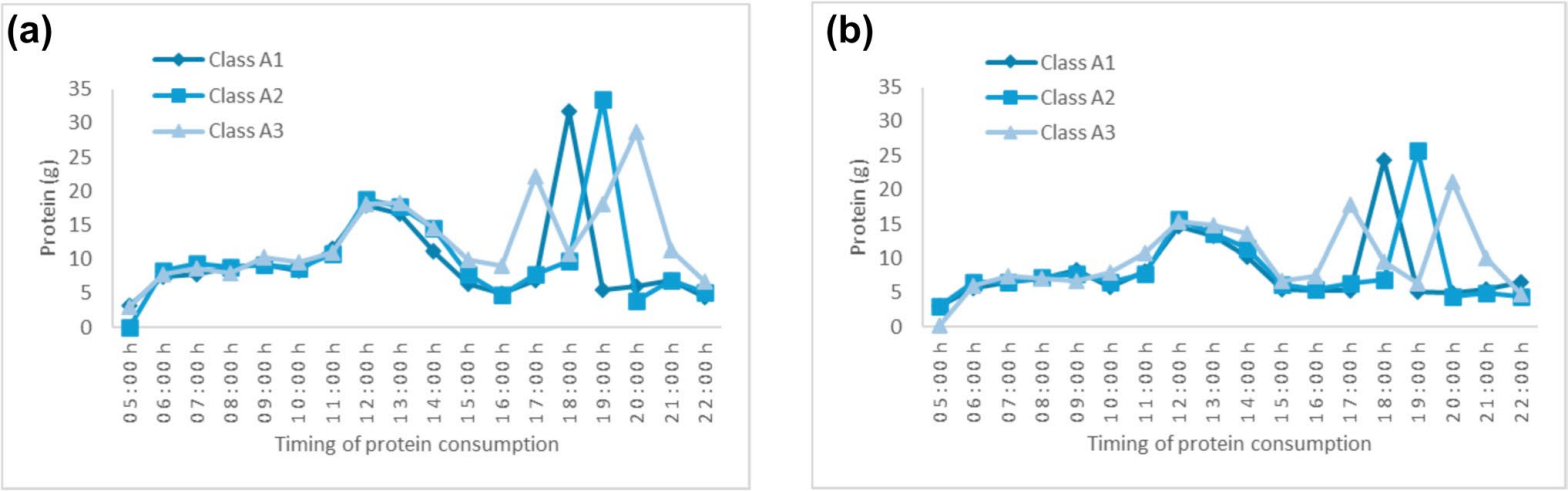
Animal protein intake of men **a** and women **b**

All temporal animal protein patterns were characterised by higher animal protein intake in the evening, albeit at different hours. (Supplementary Material 1: Fig. 2a, b and Fig. 2a, b). Men’s and women’s A1 patterns were characterised by higher conditional probabilities (> 0.9) of consuming animal protein at 18:00–19:00 h, while their A2 patterns had higher conditional probabilities for eating protein an hour later. Men and women with A3 patterns consumed animal protein either 1-h earlier (17:00–18:00 h) or 1-h later (20:00–21:00 h) than A1 patterns, as suggested by the similar probabilities for consuming animal protein at those timepoints.

The sociodemographic and eating pattern characteristics by temporal animal protein patterns are presented in Table 3. A1 patterns of both sexes were more likely to be older (*p* < 0.001) than those with A2 and A3 patterns. A higher proportion of men and women with A2 patterns had higher socioeconomic status (men *p* = 0.002, women *p* = 0.001), income and employment levels (*p* < 0.001). Compared to the A1 pattern, men and women with the A3 pattern were younger and a lower proportion were married (men *p* < 0.001, women *p* = 0.009). Men’s and women’s A3 patterns were also associated with lower EO frequency, meal frequency, and animal protein intake from meals (*p* < 0.001). The A3 pattern was also associated with higher animal protein intake from snacks among men only (*p* < 0.001).

**Table 3:** Sociodemographic characteristics of Australian men and women, by animal protein latent class membership

Characteristic	Men (N = 3169)				Women (N = 3572)			
	Class A1	Class A2	Class A3	*p*-value	Class A1	Class A2	Class A3	*p*-value
n (%)	1036 (32.7%)	943 (29.7%)	1190 (37.6%)		1226 (34.3%)	1147 (32.1%)	1199 (33.6%)	
Age [year (mean, SD)]*	52.0 (17.3)^a^	47.6 (16.4)^b^	47.6 (17.5)^b^	< 0.001	52.9 (17.7)^a^	48.1 (16.4)^b^	48.9 (18.0)^b^	< 0.001
Country of Birth [n (%)]^a^				< 0.001				< 0.001
Australia	768 (74.1%)	665 (70.6%)	777 (65.3%)		905 (73.8%)	853 (74.4%)	808 (67.4%)	
Main English-Speaking Countries (Canada, Ireland, NZ, South Africa, UK, USA)	133 (12.8%)	122 (12.9%)	175 (14.7%)		147 (12.0%)	139 (12.1%)	141 (11.8%)	
Other	135 (13.1%)	156 (16.5%)	238 (20.0%)		174 (14.2%)	155 (13.5%)	250 (20.8%)	
Geographic region				< 0.001				0.001
Major cities of Australia	657 (63.4%)	616 (65.3%)	827 (69.5%)		748 (61.0%)	734 (64.0%)	782 (65.3%)	
Inner regional Australia	236 (22.8%)	154 (16.4%)	175 (14.7%)		302 (24.6%)	222 (19.4%)	215 (17.9%)	
Other	143 (13.8%)	173 (18.3%)	188 (15.8%)		176 (14.4%)	191 (16.6%)	202 (16.8%)	
SEIFA [n (%)]				0.002				0.001
Lowest 20%	199 (19.2%)	137 (14.5%)	237 (19.9%)		251 (20.4%)	177 (15.4%)	259 (21.6%)	
Second quintile	203 (19.6%)	183 (19.4%)	248 (20.8%)		257 (21.0%)	232 (20.2%)	238 (19.8%)	
Third quintile	221 (21.3%)	176 (18.7%)	232 (19.5%)		238 (19.4%)	228 (19.9%)	249 (20.8%)	
Fourth quintile	180 (17.4%)	178 (18.9%)	213 (17.9%)		223 (18.2%)	205 (17.9%)	185 (15.4%)	
Highest 20%	233 (22.5%)	269 (28.5%)	260 (21.9%)		257 (21.0%)	305 (26.6%)	268 (22.4%)	
Income^b^				< 0.001				< 0.001
First quintile	199 (20.6%)	134 (15.1%)	206 (18.7%)		299 (26.9%)	191 (18.0%)	313 (28.7%)	
Second quintile	194 (20.1%)	117 (13.2%)	175 (15.9%)		261 (23.5%)	179 (16.8%)	224 (20.6%)	
Third quintile	207 (21.4%)	145 (16.3%)	169 (15.3%)		194 (17.4%)	193 (18.2%)	172 (15.8%)	
Fourth quintile	212 (21.9%)	212 (23.8%)	249 (22.5%)		197 (17.7%)	240 (22.5%)	195 (17.9%)	
Fifth quintile	155 (16.0%)	281 (31.6%)	305 (27.6%)		162 (14.5%)	260 (24.5%)	185 (17.0%)	
Employment				< 0.001				< 0.001
Employed	647 (62.5%)	709 (75.2%)	814 (68.4%)		597 (48.7%)	755 (65.8%)	614 (51.2%)	
Unemployed	28 (2.7%)	19 (2.0%)	34 (2.9%)		31 (2.5%)	26 (2.3%)	29 (2.4%)	
Not in the labour force	361 (34.8%)	215 (22.8%)	342 (28.7%)		598 (48.8%)	366 (31.9%)	556 (46.4%)	
Education level				0.001				< 0.001
Low	222 (21.4%)	264 (28.0%)	314 (26.4%)		254 (20.7%)	373 (32.5%)	343 (28.6%)	
Medium	446 (43.1%)	352 (37.3%)	426 (35.8%)		352 (28.7%)	333 (29.1%)	338 (28.2%)	
High	368 (35.5%)	327 (34.7%)	450 (37.8%)		620 (50.6%)	441 (38.4%)	518 (43.2%)	
Marital status				< 0.001				0.009
Married^§^	646 (62.4%)	569 (60.3%)	619 (52.0%)		635 (51.8%)	602 (52.5%)	560 (46.7%)	
Not married	390 (37.6%)	374 (39.7%)	571 (48.0%)		591 (48.2%)	545 (47.5%)	639 (53.3%)	
Household composition				< 0.001				0.016
Person living alone	252 (24.3%)	212 (22.5%)	346 (29.1%)		344 (28.1%)	305 (26.6%)	329 (27.4%)	
Couple only	369 (35.6%)	311 (33.0%)	330 (27.7%)		366 (29.9%)	303 (26.4%)	300 (25.0%)	
Couple family with children	299 (28.9%)	299 (31.7%)	337 (28.3%)		293 (23.9%)	318 (27.7%)	300 (25.0%)	
All other households	116 (11.2%)	121 (12.8%)	177 (14.9%)		223 (18.2%)	221 (19.3%)	270 (22.5%)	
Meal frequency	3.2 (0.8)^a^	3.1 (0.7)^a^	3.0 (0.9)^b^	< 0.001	3.2 (0.7)^a^	3.2 (0.7)^a^	3.1 (0.8)^b^	< 0.001
Snack frequency	1.4 (0.5)	1.5 (0.5)	1.4 (0.5)	0.82	1.4 (0.5)^a^	1.5 (0.5)^b^	1.5 (0.5)	0.002
Animal protein intake (g)	72.5 (42.7)^a^	74.4 (43.1)^a^	63.3 (42.0)^b^	< 0.001	54.5 (28.8)^a^	57.4 (30.2)^a^	46.6 (34.3)^b^	< 0.001
Animal protein intake (%EI)	12.6 (5.8)^a^	12.7 (6.5)^a^	11.3 (6.7)^b^	< 0.001	12.7 (6.4)^a^	12.7 (6.5)^a^	11.1 (7.0)^b^	< 0.001
Animal protein intake from meals (g)	65.1 (39.0)^a^	66.5 (39.9)^a^	53.8 (37.0)^b^	< 0.001	48.8 (28.1)^a^	51.3 (29.2)^a^	40.5 (32.7)^b^	< 0.001
Animal protein intake from meals (%EI)	11.4 (5.7)^a^	11.4 (6.3)^a^	9.7 (6.4)^b^	< 0.001	11.4 (6.3)^a^	11.4 (6.6)^a^	9.7 (6.9)^b^	< 0.001
Animal protein intake from snacks (g)	7.6 (12.7)^a^	8.2 (13.9)^a^	10.1 (18.2)^b^	< 0.001	5.8 (8.3)	6.3 (8.5)	6.5 (10.5)	0.17
Animal protein intake from snacks (%EI)	1.2 (1.9)^a^	1.3 (2.1)^a^	1.6 (2.7)^b^	< 0.001	1.3 (2.0)^a^	1.3 (1.7)	1.5 (2.4)^b^	0.01

^*^Differences between classes for continuous variables were assessed by using analysis of variance with Bonferroni correction, and different superscript letters indicate significant differences between classes. Values are weighted means (SDs)

^a^Differences between classes for categorical variables were assessed by using adjusted Pearson’s chi-square test. Values are weighted percentages

^b^n = 2960 men and n = 3265 women due to missing cases for income

^§^A registered or de facto marriage

### Latent classes of temporal plant protein patterns

**Fig. 3:**
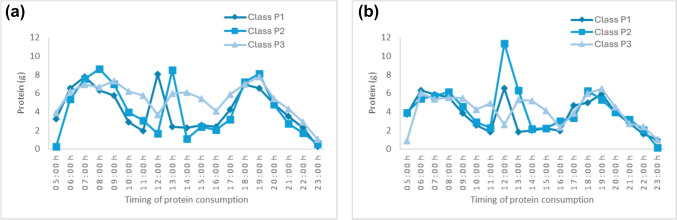
Plant protein intake of men **a** and women **b**

Temporal plant protein patterns varied by conditional probabilities for consuming plant protein in the middle of the day. Men’s and women’s P1 patterns were characterised by higher probabilities of consuming plant protein at 12:00–13:00 h, while P2 patterns of both sexes had higher probabilities for eating protein an hour later. Men and women with the P3 pattern consumed plant protein either 1-h earlier (11:00–12:00 h) or 1-h later (14:00–15:00 h). All patterns had a similar distribution of intake amount across the day (Supplementary Material 1: Fig.3 a, b and Fig. 3a, b), except for a small proportion of women with P2 pattern who had a higher amount of plant protein at 12:00 h (Additional File 2). For example, the average plant protein intake of men’s P1 and P2 patterns was 8.5–8.7 g at 07:00–09:00 h (peak hours in the morning), 8.8–9.1 g at 12:00–14:00 h (peak hours in the midday), and 8.2–8.9 g at 18:00–20:00 h (peak hours in the evening).

The sociodemographic and eating pattern characteristics by temporal plant protein patterns are presented in Table 4. P1 patterns were more likely to be older (women only, *p* < 0.001) than those with P2 and P3 patterns. A higher proportion of men and women with P2 patterns had higher socioeconomic status (*p* = 0.001), but only women’s P2 pattern was significantly associated with higher income (*p* = 0.02) and employment levels (*p* < 0.001). Men and women with the P3 pattern were younger than those with the P1 pattern (*p* < 0.001), and men’s P3 pattern had a lower proportion of married adults (*p* < 0.001). Men’s and women’s P3 patterns were associated with lower EO frequency, meal frequency, and plant protein intake from meals (*p* < 0.001), but higher plant protein intake from snacks (*p* < 0.001).

**Table 4:** Sociodemographic characteristics of Australian men and women, by plant protein latent class membership

Characteristic	Men (N = 3169)				Women (N = 3572)			
	Class P1	Class P2	Class P3	*p*-value	Class P1	Class P2	Class P3	*p*-value
n(%)	1243 (39.2%)	807 (25.5%)	1119 (35.3%)		1347 (37.7%)	1105 (30.9%)	1120 (31.4%)	
Age [year (mean, SD)]*	51.0 (17.4)^a^	49.5 (17.2)^a^	46.5 (16.8)^b^	< 0.001	52.2 (18.1)^a^	49.6 (17.2)^b^	47.8 (16.9)^b^	< 0.001
Country of Birth [n (%)]^a^				0.46				0.001
Australia	880 (70.8%)	566 (70.1%)	764 (68.3%)		1,005(74.6%)	803 (72.7%)	758 (67.7%)	
Main English-Speaking Countries (Canada, Ireland, NZ, South Africa, UK, USA)	172 (13.8%)	107 (13.3%)	151 (13.5%)		143 (10.6%)	139 (12.6%)	145 (12.9%)	
Other	191 (15.4%)	134 (16.6%)	204 (18.2%)		199 (14.8%)	163 (14.7%)	217 (19.4%)	
Geographic region				0.001				0.026
Major cities of Australia	822 (66.1%)	553 (68.5%)	725 (64.8%)		819 (60.8%)	715 (64.7%)	730 (65.2%)	
Inner regional Australia	209 (16.8%)	162 (20.1%)	194 (17.3%)		300 (22.3%)	235 (21.3%)	204 (18.2%)	
Other	212 (17.1%)	92 (11.4%)	200 (17.9%)		228 (16.9%)	155 (14.0%)	186 (16.6%)	
SEIFA [n (%)]				< 0.001				< 0.001
Lowest 20%	219 (17.6%)	111 (13.8%)	243 (21.7%)		264 (19.6%)	179 (16.2%)	244 (21.8%)	
Second quintile	257 (20.7%)	159 (19.7%)	218 (19.5%)		311 (23.1%)	195 (17.6%)	221 (19.7%)	
Third quintile	247 (19.9%)	160 (19.8%)	222 (19.8%)		257 (19.1%)	232 (21.0%)	226 (20.2%)	
Fourth quintile	222 (17.8%)	143 (17.7%)	206 (18.4%)		213 (15.8%)	207 (18.8%)	193 (17.2%)	
Highest 20%	298 (24.0%)	234 (29.0%)	230 (20.6%)		302 (22.4%)	292 (26.4%)	236 (21.1%)	
Income^b^				0.28				0.02
First quintile	219 (18.6%)	119 (15.8%)	201 (19.5%)		315 (25.8%)	220 (21.7%)	268 (26.0%)	
Second quintile	192 (16.3%)	112 (14.9%)	182 (17.6%)		274 (22.5%)	189 (18.6%)	201 (19.5%)	
Third quintile	213 (18.1%)	138 (18.3%)	170 (16.5%)		199 (16.3%)	190 (18.7%)	170 (16.5%)	
Fourth quintile	271 (23.1%)	179 (23.8%)	223 (21.6%)		224 (18.4%)	202 (19.9%)	206 (20.0%)	
Fifth quintile	280 (23.9%)	205 (27.2%)	256 (24.8%)		207 (17.0%)	214 (21.1%)	186 (18.0%)	
Employment				0.24				< 0.001
Employed	836 (67.3%)	561 (69.5%)	773 (69.1%)		668 (49.6%)	663 (60.0%)	635 (56.7%)	
Unemployed	30 (2.4%)	15 (1.9%)	36 (3.2%)		17 (1.3%)	29 (2.6%)	40 (3.6%)	
Not in the labour force	377 (30.3%)	231 (28.6%)	310 (27.7%)		662 (49.1%)	413 (37.4%)	445 (39.7%)	
Education level				< 0.001				0.001
Low	311 (25.0%)	246 (30.5%)	243 (21.7%)		350 (26.0%)	321 (29.0%)	299 (26.7%)	
Medium	468 (37.7%)	294 (36.4%)	462 (41.3%)		346 (25.7%)	333 (30.1%)	344 (30.7%)	
High	464 (37.3%)	267 (33.1%)	414 (37.0%)		651 (48.3%)	451 (40.8%)	477 (42.6%)	
Marital status				< 0.001				0.068
Married^§^	766 (61.6%)	514 (63.7%)	554 (49.5%)		701 (52.0%)	564 (51.0%)	532 (47.5%)	
Not married	477 (38.4%)	293 (36.3%)	565 (50.5%)		646 (48.0%)	541 (49.0%)	588 (52.5%)	
Household composition				< 0.001				< 0.001
Person living alone	310 (24.9%)	172 (21.3%)	328 (29.3%)		392 (29.1%)	301 (27.2%)	285 (25.4%)	
Couple only	438 (35.2%)	259 (32.1%)	313 (28.0%)		386 (28.7%)	303 (27.4%)	280 (25.0%)	
Couple family with children	361 (29.0%)	280 (34.7%)	294 (26.3%)		321 (23.8%)	304 (27.5%)	286 (25.5%)	
All other households	134 (10.8%)	96 (11.9%)	184 (16.4%)		248 (18.4%)	197 (17.8%)	269 (24.0%)	
EO frequency	4.7 (0.8)^a^	4.6 (0.9)^a^	4.3 (1.0)^b^	< 0.001	4.7 (0.8)^a^	4.7 (0.8)^a^	4.4 (1.0)^b^	< 0.001
Meal frequency	3.2 (0.7)^a^	3.2 (0.7)^a^	2.8 (0.9)^b^	< 0.001	3.3 (0.7)^a^	3.3 (0.7)^a^	2.9 (0.9)^b^	< 0.001
Snack frequency	1.4 (0.5)	1.4 (0.5)^a^	1.5 (0.5)^b^	0.04	1.5 (0.5)	1.5 (0.5)	1.5 (0.5)	0.39
Plant protein intake (g)	33.5 (16.0)^a^	34.1 (15.6)^a^	31.6 (19.9)^b^	0.004	27.4 (12.5)^a^	27.3 (12.9)^a^	24.1 (13.5)^b^	< 0.001
Plant protein intake (%EI)	5.8 (2.1)^a^	5.9 (2.1)^a^	5.4 (2.2)^b^	< 0.001	6.2 (2.1)^a^	6.2 (2.2)^a^	5.6 (2.4)^b^	< 0.001
Plant protein intake from meals (g)	27.9 (13.9)^a^	29.1 (14.3)^a^	23.7 (13.4)^b^	< 0.001	22.9 (11.2)^a^	22.8 (11.3)^a^	18.9 (11.4)^b^	< 0.001
Plant protein intake from meals (%EI)	4.9 (2.1)^a^	5.1 (2.2)^a^	4.2 (2.2)^b^	< 0.001	5.2 (2.0)^a^	5.2 (2.1)^a^	4.5 (2.3)^b^	< 0.001
Plant protein intake from snacks (g)	5.8 (7.0)^a^	5.2 (6.4)^a^	8.3 (14.1)^b^	< 0.001	4.6 (5.3)^a^	4.6 (5.6)^a^	5.4 (7.1)^b^	< 0.001
Plant protein intake from snacks (%EI)	0.9 (0.9)^a^	0.9 (0.9)^a^	1.3 (1.4)^b^	< 0.001	1.0 (1.0)^a^	1.0 (1.2)^a^	1.2 (1.4)^b^	< 0.001

^*^Differences between classes for continuous variables were assessed by using analysis of variance with Bonferroni correction, and different superscript letters indicate significant differences between classes. Values are weighted means (SDs)

^a^Differences between classes for categorical variables were assessed by using adjusted Pearson’s chi-square test. Values are weighted percentages

^b^n = 2960 men and n = 3265 women due to missing cases for income

^§^A registered or de facto marriage

## Discussion

The present study identified three distinct temporal patterns of total, animal, and plant protein intakes in Australian adults, which is significant because the diversity of these patterns may reflect different sociocultural motivations or beliefs that can serve as drivers or barriers for adopting more sustainable diets (e.g., plant-based diets) [32] and adhering to healthy eating styles (e.g., intermittent fasting) [33]. In terms of total protein intake, all mean intakes fell within the macronutrient distribution ranges for protein (15–25%) [29], but towards the lower end. Regardless of patterns, a large proportion of total and animal proteins were consumed in the evening, while plant protein was most likely eaten at midday with the amount of intake being similar across mealtimes. Sociodemographic and eating pattern characteristics varied between patterns. In most patterns, men and women with lower EO and meal frequencies were more likely to have lower protein intake from meals but higher protein intake from snacks.

### Temporal distribution of protein intake

Australians had most of their protein intake in the evening (after 17:00 h), with animal protein being the main source and plant protein intake being more equally distributed throughout the day, regardless of the temporal patterns. The finding regarding a large proportion of total protein intake in the evening is similar to previous observational studies in comparable countries to Australia (United States, Great Britain, and New Zealand) suggesting adults have the largest proportion of their total protein intake in the evening, [7, 34, 35] which aligns with the evening meal being the major meal in many Western countries [36]. The present study also found that Australian protein intake was predominantly from animal sources, with the largest intake also in the evening, which aligns with previous observational studies suggesting that animal-source foods constitute a major protein source for Dutch, New Zealander, and Irish adults [5, 35, 37]. In terms of temporal plant protein distribution, the present study also aligns with previous studies suggesting a similar amount of intake across the day [5, 35, 37]. For example, the average plant protein intake of New Zealander adults in the morning, midday, and evening reported in the previous study was 9.5 g, 10.8 g, and 11.8 g, respectively and is similar to the plant protein intakes observed for the first and second class in the present study [35].

There are only a few studies have examined temporal protein patterns making comparison difficult. A study by Lucassen et al. [38] assessed the timing of eating and protein intake of adults grouped by their chronotype which describes the body’s preference to sleep and wake at certain times and can influence appetite and eating behaviour [17]. They found an 80-min difference in eating time, mainly at breakfast, probably reflecting different waking times between adults with morning-type and evening-type [38]. Another study grouping Finnish adults by their chronotype also found that adults with evening type consistently had their peak energy and macronutrient intake an hour later than those with morning type [38], which is similar to the patterns we identified as T1 and T2. However, as our study did not include measures of chronotype therefore we are unable to identify whether these patterns are similar due to overlapping chronotype. Also, among these small number of studies, relatively small and inconsistent differences have been identified but the importance of these small differences in timing of protein intake is unclear.

### Characteristics of temporal protein patterns

The difference in eating timing between the temporal protein patterns observed in the present study may be influenced by sociodemographic factors. For example, T2, A2, and P2 patterns of men and women whose protein consumption was likely to be 1-h later than T1, A1 (in the evening), and P1 patterns (in midday) had higher income, socioeconomic, and employment status. This somewhat aligns with previous British studies suggesting that protein intake timing shifted toward a later time in the day (at 11:00–16:00 h and 16:00–23:59 h) [34], and the higher intake later in the evening was associated with higher social class and non-manual occupations (e.g., managerial and skilled professional workers) [39]. Furthermore, the difference between source-specific protein patterns might be explained by age and household structures, in which protein intake, eating and meal frequencies are more likely to be higher in married, older adults, and couples with and without children. In this study, men and women with A1-A2 and P1-P2 patterns who had a higher proportion of animal and plant protein from meals were older and had a higher proportion of married adults and couples with or without children when compared to adults with A3 and P3 patterns which were characterised by a lower proportion of animal and plant protein from meals. This is probably because commensality or family structures might contribute to higher intake of protein-containing foods and more eating and meal frequencies [36].

In contrast to Class A1 and Class P1, the findings on Class A3 and P3 of both sexes having lower intake, EO and meal frequency support previous studies suggesting that men and women are more likely to have lower protein intake as the number of EOs and meals decrease [18, 40]. Interestingly, Class A3 (men only) and P3 (both sexes) had higher amounts of protein intake from snacking occasions compared to the other classes, which is potentially related to the fact that younger adults are more likely to snack and skip meals- due to time scarcity, changing social commitments, and transition to a new social environment [17, 39, 41].

Unlike meal frequency which tends to be proportionate with the amount consumed [40], we found variability in the snacking frequency and the amount of protein intake from snacking in each pattern. For example, we observed different amounts of protein consumed from snacks despite the same snack frequency in men (Class A3 vs. A1-A2) and women (Class P3 vs. P1-P2). This could be because snacks are culturally seen as unstructured EOs with higher variability in both frequency and food types of individuals across the day [36]. This finding might also be influenced by the different snack preferences of adults in each class, where many preferred snacks high in carbohydrates but low in protein, as suggested in previous studies [41, 42].

### Strengths and limitations

To our knowledge, this study is the first to assess temporal protein patterns of Australian adults using a data-driven method. This study also provides further understanding of protein intake distribution throughout the day by assessing the patterns of animal and plant protein intake separately, which may differentially influence the associations between protein intake and health. Another strength includes the identification of sociodemographic and eating pattern characteristics, which explains the distinction between three temporal protein patterns.

Some limitations should be considered in this study. Despite the nationally representative sample, the analysis used survey data from 2011–2012, and the data from 12 years ago may not represent current intake patterns. The COVID-19 pandemic might have also changed Australians’ work and commute hours [43], which could affect protein intake timing, and therefore warrant further analysis once more recent survey data has been published. This study used 1-d dietary recall data and was, therefore, unable to capture the potential day-to-day variability in protein intake patterns, especially between weekdays and weekend days. However, current methods used to model usual dietary intake are designed to estimate total daily intake and could not be applied to our study, which examined temporal intake at eating occasions. Another limitation is the generalisability of Australian dietary patterns and context may be different from other countries.

Moreover, this study used self-reported time of eating, which warrants future studies with more advanced measures of timing, such as picture-based or wearable technologies [4]. The other limitation is the lack of data on factors that potentially influence intake timing, such as different work patterns, occupational status (hours, schedules, and flexibility), and sleep timing and quality [17], which could be further explored in future studies. Another limitation is the cross-sectional design of this study limiting causality, which may warrant further investigations with RCTs considering intake timing of protein and other macronutrients. Lastly, despite the identified age difference between patterns, this study did not separate younger and older adults whose protein intake and requirements might be different. Future sex- and age-stratified patterns may be important for other outcomes, such as examining the impact of temporal protein patterns on ageing-related health outcomes.

### Future research/implications

The present study’s findings on temporal protein patterns suggest that how and when people eat total, animal and plant protein varies across population groups with different sociodemographic and eating pattern characteristics. The emerging concepts of eating timing and different eating styles (e.g., intermittent fasting) have been suggested to be relevant for integration into dietary guidelines [4], due to their important roles in body weight and cardiometabolic health [44]. However, the present study did not include measures of cardiometabolic health (e.g., obesity and glycaemic measures), and further research, including randomised controlled trials, examining the different health implications of temporal protein patterns may inform protein intake timing recommendations for use in dietary guidelines. To date, the available evidence on the associations between temporal total protein patterns and cardiometabolic health is mixed, mainly due to the heterogeneity in measuring eating timing and regularity [8]. The inconsistency has also been reported in associations with muscle health, particularly when comparing even vs. skewed temporal protein distribution [45], and therefore, a more standardised approach to measuring eating timing is needed to allow the comparison of results.

It is also worth noting that the present study focuses on protein in isolation, so it does not capture the whole meal containing other foods and macronutrients. Nonetheless, the overall temporal patterning of foods and balance of macronutrients across the day is also likely to be important for health. For example, previous evidence suggested the impact of carbohydrate and fat intake timing on glycaemic control, mainly among adults with impaired glucose metabolism [46]. Future dietary recommendations may also benefit from further investigations on associations between temporal protein patterns and diet quality, given that protein intake is sourced from a variety of foods (e.g. refined grain vs whole grain cereals, lean cuts of meat and poultry vs processed meat high in fat and salt) [47] that may differentially influence overall diet quality [48, 49].

### Conclusion

This study identified three distinct temporal patterns of total, plant, and animal protein intake in Australian adults, which have different sociodemographic and eating pattern characteristics. Future research needs to investigate whether temporal protein patterns are associated with diet quality and health outcomes.

## Supplementary Information

Below is the link to the electronic supplementary material.

Supplementary file1 (PDF 121 KB)

Supplementary file2 (PDF 195 KB)

## Data Availability

Data are available on request. The authors do not have permission to release the data to third parties. Permission can be applied from the Australian Bureau of Statistics (https://secure.abs.gov.au/micro/protected/pages/home.xhtml).
