# Supplementary material for: Temporal patterns of total, animal and plant protein intakes of Australian adults: a latent class analysis
Source: Eur J Nutr. 2026 Feb 12;65(2):50. doi: 10.1007/s00394-026-03918-8 (PMC12901164; doi:10.1007/s00394-026-03918-8)
Supplement: Supplementary file 1 — Supplementary file1 (PDF 121 KB) [file 394_2026_3918_MOESM1_ESM.pdf]

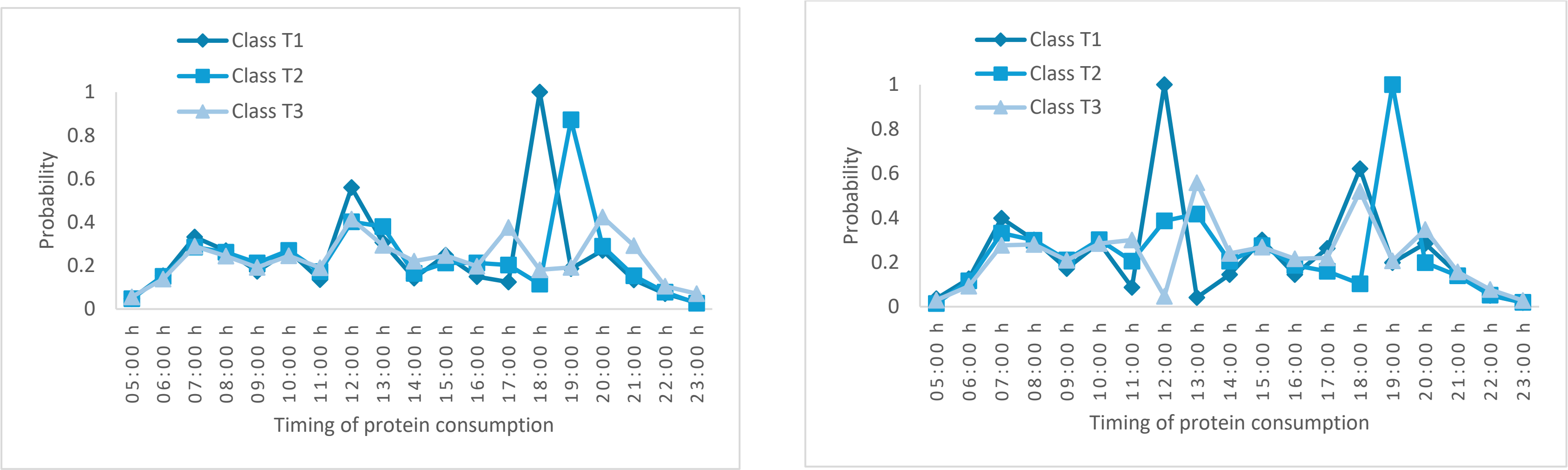

Supplementary figure 1a-1b. Conditional probabilities of total protein intake at eating occasion in men (1a) and women (1b)

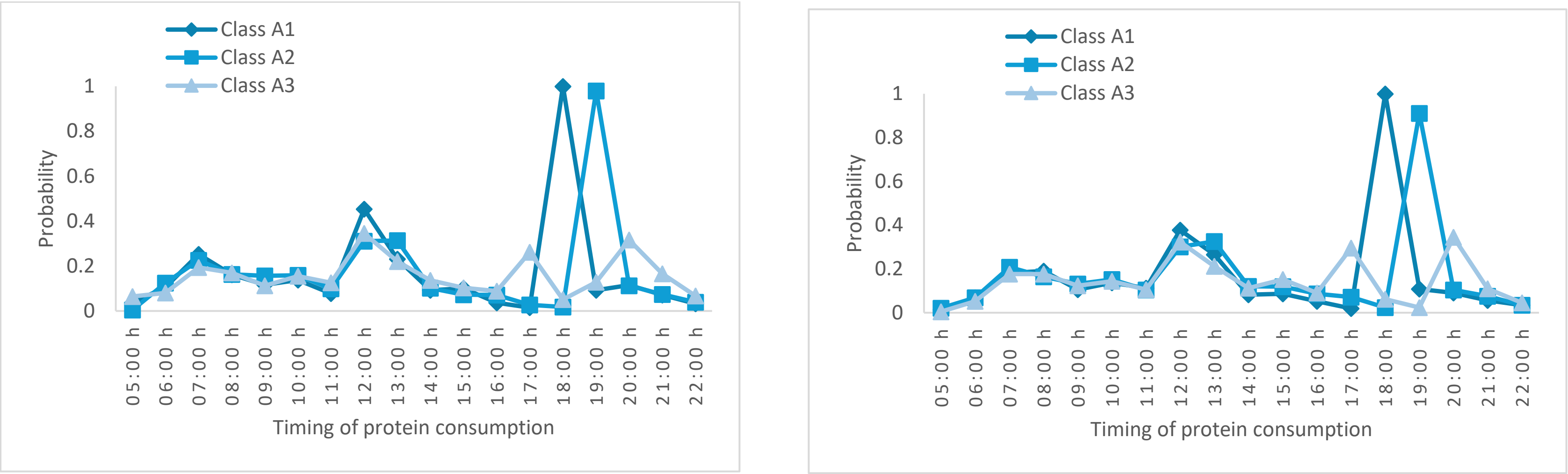

Supplementary figure 2a-2b. Conditional probabilities of animal protein intake at eating occasion in men (2a) and women (2b)

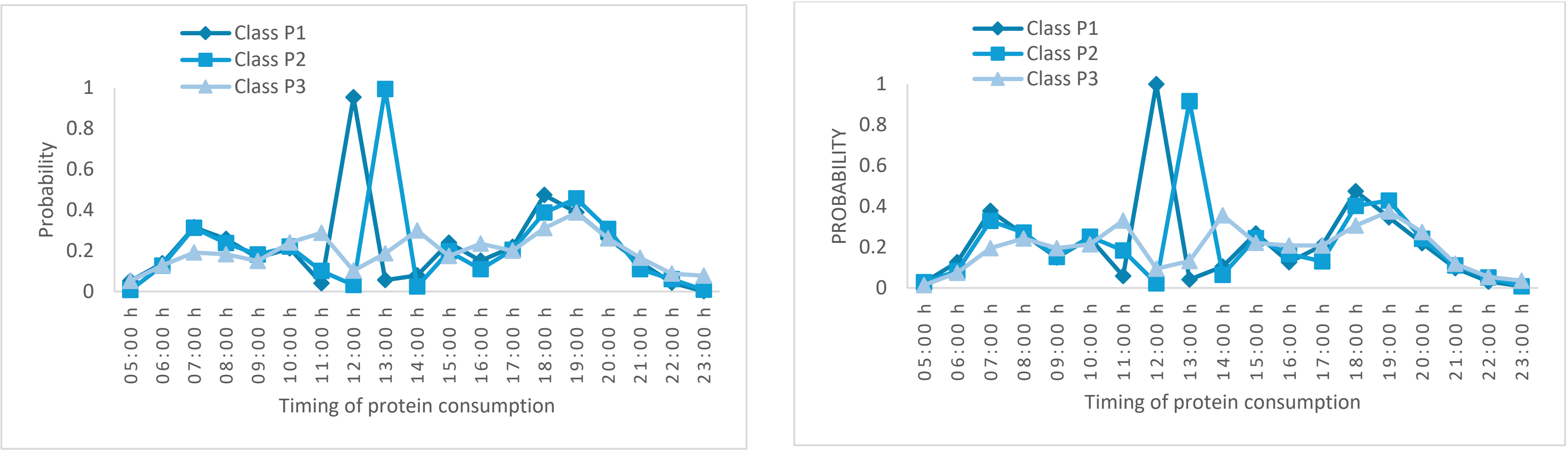

Supplementary figure 3a-3b. Conditional probabilities of plant protein intake at eating occasion in men (3a) and women (3b)
