# Supplementary material for: Temporal patterns of total, animal and plant protein intakes of Australian adults: a latent class analysis
Source: Eur J Nutr. 2026 Feb 12;65(2):50. doi: 10.1007/s00394-026-03918-8 (PMC12901164; doi:10.1007/s00394-026-03918-8)
Supplement: Supplementary file 2 — Supplementary file2 (PDF 195 KB) [file 394_2026_3918_MOESM2_ESM.pdf]

Supplementary table 1. Hourly protein intake according to latent protein classes of Australian men

Time of day	Class 1		Class 2		Class 3	
	Percentage (%) of consumers	Mean (95% CI) intake (g) among consumers	Percentage (%) of consumers	Mean (95% CI) intake (g) among consumers	Percentage (%) of consumers	Mean (95% CI) intake (g) among consumers
**Total protein***						
05:00h	4.6	6.7 (6.0, 7.4)	4.8	7.7 (7.2 8.1)	4.5	6.4 (5.5, 7.3)
06:00h	15.7	12.3 (11.3, 13.3)	15.5	13.1 (12.1, 14.1)	13.0	12.2 (11.0, 13.4)
07:00h	31.4	15.0 (14.0, 16.0)	28.0	15.7 (14.7,16.7)	28.3	14.2 (13.1, 15.4)
08:00h	28.1	14.1 (13.1, 15.1)	26.1	13.7 (12.7,14.7)	25.3	13.5 (12.3, 14.7)
09:00h	19.6	13.1 (11.8, 14.4)	21.9	13.6 (12.5,14.8)	19.7	14.8 (13.3, 16.2)
10:00h	27.5	10.2 (9.2, 11.3)	27.6	10.7 (9.6,11.8)	24.8	11.6 (10.3, 12.9)
11:00h	15.7	12.6 (10.9, 14.2)	17.9	12.4 (10.8,13.9)	20.5	15.1 (13.5, 16.7)
12:00h	54.1	24.9 (23.4, 26.4)	40.0	25.8 (24.1,27.4)	39.3	25.2 (23.2, 27.1)
13:00h	30.8	22.6 (20.8, 24.4)	39.0	25.2 (23.5,26.8)	31.8	24.2 (22.2, 26.3)
14:00h	14.9	12.8 (10.7, 15.0)	17.1	15.1 (13.1,17.0)	20.6	17.8 (15.6, 19.9)
15:00h	24.6	5.7 (4.9, 6.5)	23.3	7.4 (6.5,8.3)	23.8	9.8 (8.6, 11.0)
16:00h	14.3	6.0 (5.0, 6.9)	17.5	6.1 (5.3,7.0)	21.7	9.6 (8.6, 10.7)
17:00h	12.2	4.0 (3.0, 4.9)	18.6	13.3 (11.0,15.5)	38.5	26.1 (24.0, 28.1)
18:00h	100	43.3 (41.9, 44.7)	10.3	4.8 (3.7,5.9)	15.1	6.6 (5.2, 8.0)
19:00h	18.0	4.8 (4.0, 5.6)	92.7	43.6 (42.2,45.1)	15.4	5.6 (4.5, 6.8)
20:00h	25.3	5.2 (4.7, 5.8)	27.3	4.8 (4.3,5.3)	43.5	44.0 (42.4, 45.5)
21:00h	14.2	8.0 (6.7, 9.3)	15.6	7.5 (6.3,8.7)	26.6	13.8 (12.4, 15.3)
22:00h	7.2	5.5 (4.6, 6.4)	8.9	5.8 (5.0,6.6)	11.2	8.0 (7.2, 8.8)
23:00h	3.1	3.5 (2.9, 4.1)	2.7	3.8 (3.2,4.4)	7.5	3.7 (3.3, 4.1)
**Animal protein†**						
05:00h	3.1	3.1 (3.1, 3.1)	0.5	0.2 (-0.2, 0.6)	5.7	3.1 (3.0, 3.1)
06:00h	10.6	7.5 (6.9, 8.1)	11.5	8.4 (7.8, 9.0)	8.6	8.1 (7.4, 8.8)
07:00h	22.5	9.3 (8.5, 10.1)	21.6	10.2 (9.4, 11.0)	17.8	10.1 (9.3, 11.0)
08:00h	18.3	9.8 (8.9, 10.6)	17.3	9.6 (8.7, 10.6)	17.4	9.6 (8.8, 10.4)
09:00h	12.9	10.5 (9.3, 11.6)	15.8	11.1 (10.0, 12.1)	12.2	11.6 (10.5, 12.6)
10:00h	14.1	10.1 (9.0, 11.1)	16.4	10.4 (9.3, 11.5)	15.0	10.6 (9.6, 11.6)
11:00h	9.1	13.2 (11.5, 15.0)	11.1	12.3 (10.8, 13.9)	13.4	13.1 (11.7, 14.4)
12:00h	42.5	21.7 (20.1, 23.2)	31.0	22.6 (20.7, 24.5)	31.8	21.0 (19.3, 22.6)
13:00h	22.6	19.2 (17.4, 21.0)	31.8	21.0 (19.4, 22.7)	23.5	22.0 (20.2, 23.7)
14:00h	8.9	13.6 (11.3, 15.9)	10.9	14.6 (12.6, 16.7)	12.4	17.0 (15.2, 18.8)

15:00h	9.2	7.1 (5.9, 8.3)	9.2	9.1 (7.8, 10.3)	10.2	10.7 (9.6, 11.8)
16:00h	4.6	6.9 (5.5, 8.3)	5.9	6.9 (5.5, 8.3)	9.7	9.8 (9.0, 10.7)
17:00h	2.0	7.7 (4.2, 11.2)	2.1	8.4 (4.9, 11.9)	27.6	25.8 (24.0, 27.5)
18:00h	100.0	35.6 (34.2, 36.9)	1.7	11.0 (3.7, 18.3)	2.7	10.3 (6.2, 14.4)
19:00h	9.1	6.0 (4.7, 7.2)	99.9	36.1 (34.6, 37.5)	6.5	22.5 (17.1, 27.9)
20:00h	11.6	5.8 (4.8, 6.7)	9.9	4.8 (4.0, 5.6)	36.0	32.9 (31.2, 34.5)
21:00h	7.7	7.9 (6.6, 9.2)	8.5	6.8 (5.6, 8.0)	16.1	12.6 (11.5, 13.7)
22:00h	3.8	5.9 (4.8, 7.0)	4.8	6.2 (5.3, 7.1)	7.1	7.4 (6.9, 8.0)
			**Plant protein**‡			
05:00h	4.6	3.5 (3.3, 3.8)	0.6	0.5 (0.0, 1.1)	5.5	3.7 (3.4, 3.9)
06:00h	15.8	7.2 (6.8, 7.7)	12.1	7.0 (6.2, 7.8)	11.3	6.9 (6.2, 7.7)
07:00h	31.8	8.5 (8.0, 9.0)	28.3	8.3 (7.6, 8.9)	19.8	7.8 (7.1, 8.4)
08:00h	24.9	7.4 (6.9, 7.9)	28.4	8.7 (8.2, 9.3)	18.1	7.2 (6.6, 7.8)
09:00h	17.8	6.4 (5.9, 6.9)	19.2	7.1 (6.4, 7.7)	15.7	7.9 (7.2, 8.5)
10:00h	21.0	3.6 (3.2, 4.0)	21.9	4.7 (4.2, 5.3)	25.6	7.0 (6.5, 7.4)
11:00h	4.7	2.1 (1.5, 2.6)	10.4	3.2 (2.6, 3.8)	30.4	6.5 (6.1, 6.9)
12:00h	98.9	8.8 (8.5, 9.1)	3.0	1.8 (1.2, 2.5)	8.0	4.4 (3.5, 5.2)
13:00h	5.0	3.4 (2.6, 4.3)	99.8	9.1 (8.8, 9.5)	13.6	7.1 (6.2, 8.0)
14:00h	7.8	2.8 (2.2, 3.3)	3.0	0.9 (0.7, 1.2)	31.9	6.7 (6.3, 7.1)
15:00h	23.3	3.4 (3.1, 3.8)	19.7	2.8 (2.4, 3.2)	19.3	5.7 (5.2, 6.1)
16:00h	13.4	3.3 (2.9, 3.6)	11.0	2.1 (1.8, 2.5)	22.4	4.7 (4.4, 5.0)
17:00h	22.0	5.4 (4.8, 5.9)	18.3	4.7 (4.0, 5.4)	19.7	6.7 (6.0, 7.3)
18:00h	47.1	8.2 (7.7, 8.7)	38.9	8.5 (7.8, 9.2)	32.4	8.5 (7.8, 9.2)
19:00h	38.1	8.3 (7.7, 8.9)	45.6	8.9 (8.2, 9.6)	37.9	9.0 (8.4, 9.6)
20:00h	25.8	5.9 (5.3, 6.5)	28.5	6.1 (5.4, 6.8)	25.6	6.9 (6.3, 7.5)
21:00h	13.4	4.0 (3.6, 4.5)	13.4	3.5 (2.9, 4.1)	15.4	4.9 (4.4, 5.4)
22:00h	5.0	2.3 (2.0, 2.7)	6.1	2.1 (1.7, 2.4)	8.5	2.9 (2.6, 3.1)
23:00h	0.2	0.3 (0.1, 0.4)	0.9	0.5 (0.5, 0.6)	8.2	1.0 (1.0, 1.0)

*Class 1, 2, and 3 of total protein correspond to Class T1, T2, and T3, respectively.

†Class 1, 2, and 3 of animal protein correspond to Class A1, A2, and A3, respectively.

‡Class 1, 2, and 3 of plant protein correspond to Class P1, P2, and P3, respectively.

Supplementary table 2. Hourly protein intake according to latent protein classes of Australian women

Time of day	Class 1		Class 2		Class 3	
	Percentage (%) of consumers	Mean (95% CI) intake (g) among consumers	Percentage (%) of consumers	Mean (95% CI) intake (g) among consumers	Percentage (%) of consumers	Mean (95% CI) intake (g) among consumers
**Total protein***						
05:00h	3.8	5.9 (5.0, 6.8)	1.8	5.3 (3.8, 6.8)	3.1	5.1 (4.2, 5.9)
06:00h	12.8	11.2 (10.2, 12.2)	11.1	10.1 (9.0, 11.2)	9.3	10.0 (8.8, 11.1)
07:00h	37.7	11.1 (10.4, 11.9)	31.7	11.6 (10.8, 12.4)	26.5	10.6 (9.9, 11.4)
08:00h	30.0	10.4 (9.6, 11.1)	29.8	11.1 (10.3, 12.0)	28.7	10.8 (10.1, 11.5)
09:00h	17.2	8.4 (7.5, 9.2)	20.5	10.1 (9.0, 11.1)	22.2	11.0 (10.1, 11.9)
10:00h	29.0	5.8 (5.1, 6.4)	30.0	7.6 (6.8, 8.5)	27.3	8.0 (7.2, 8.7)
11:00h	9.5	6.4 (4.9, 7.9)	20.9	8.7 (7.6, 9.9)	31.4	10.8 (9.9, 11.7)
12:00h	100.0	20.7 (19.8, 21.7)	38.3	20.8 (19.3, 22.4)	2.5	8.4 (5.5, 11.2)
13:00h	4.0	3.2 (2.1, 4.2)	41.8	19.6 (18.2, 21.0)	58.7	19.8 (18.8, 20.8)
14:00h	14.5	5.0 (4.0, 5.9)	20.5	13.7 (12.1, 15.4)	23.7	14.0 (12.6, 15.4)
15:00h	29.0	5.2 (4.5, 5.9)	24.9	6.2 (5.4, 7.1)	24.2	7.6 (6.8, 8.4)
16:00h	16.4	5.2 (4.3, 6.0)	19.7	4.8 (4.1, 5.5)	20.1	6.8 (6.0, 7.6)
17:00h	27.3	22.2 (20.1, 24.2)	14.4	4.6 (3.5, 5.8)	22.4	18.1 (16.2, 20.0)
18:00h	63.7	30.3 (28.7, 31.9)	10.7	3.9 (3.0, 4.8)	54.3	30.4 (28.8, 31.9)
19:00h	19.9	5.6 (4.9, 6.2)	100.0	39.3 (38.1, 40.5)	19.0	5.4 (4.9, 6.0)
20:00h	29.0	14.6 (12.7, 16.5)	19.9	5.3 (4.4, 6.3)	34.6	20.5 (18.8, 22.3)
21:00h	15.3	10.4 (8.8, 12.0)	14.2	4.6 (3.8, 5.3)	16.5	9.2 (7.9, 10.5)
22:00h	4.7	5.9 (4.7, 7.1)	5.8	4.9 (3.9, 5.8)	7.6	6.1 (5.3, 6.9)
23:00h	2.5	3.7 (3.0, 4.4)	2.3	3.7 (3.0, 4.4)	2.9	3.6 (3.1, 4.1)
**Animal protein†**						
05:00h	1.5	3.1 (2.9, 3.2)	2.6	3.0 (2.9, 3.2)	0.2	0.1 (0.1, 0.1)
06:00h	6.8	6.4 (5.6, 7.2)	6.8	7.4 (6.6, 8.2)	5.3	7.1 (6.2, 7.9)
07:00h	18.6	7.9 (7.3, 8.6)	19.7	8.1 (7.4, 8.8)	17.8	8.2 (7.4, 9.0)
08:00h	18.8	7.7 (7.1, 8.4)	16.4	8.6 (7.8, 9.4)	16.7	8.3 (7.5, 9.0)
09:00h	10.5	8.6 (7.6, 9.5)	13.3	8.5 (7.5, 9.5)	12.4	8.2 (7.3, 9.1)
10:00h	13.1	7.2 (6.4, 8.0)	15.1	7.9 (7.1, 8.8)	13.5	8.0 (7.2, 8.9)
11:00h	11.8	10.1 (8.8, 11.4)	10.6	9.8 (8.4, 11.1)	11.6	11.1 (9.8, 12.3)
12:00h	37.4	16.7 (15.4, 17.9)	30.1	18.3 (16.8, 19.8)	31.4	18.8 (17.2, 20.5)
13:00h	26.1	16.4 (15.0, 17.9)	31.4	17.2 (15.8, 18.6)	24.6	18.0 (16.5, 19.6)
14:00h	8.7	11.1 (9.4, 12.8)	12.9	12.7 (11.2, 14.3)	11.4	15.2 (13.5, 17.0)

15:00h	9.2	6.8 (5.8, 7.8)	11.0	7.5 (6.5, 8.5)	13.1	8.3 (7.3, 9.3)
16:00h	5.6	6.4 (5.1, 7.6)	7.8	5.9 (4.9, 6.9)	10.1	7.9 (7.0, 8.8)
17:00h	1.8	4.0 (2.4, 5.6)	6.5	11.5 (8.7, 14.4)	30.3	23.2 (21.6, 24.8)
18:00h	100.0	28.1 (27.0, 29.2)	1.8	6.9 (4.2, 9.7)	3.5	8.5 (5.2, 11.8)
19:00h	10.1	5.8 (4.7, 6.9)	98.5	29.1 (27.9, 30.2)	1.0	3.5 (2.0, 4.9)
20:00h	10.6	5.6 (4.8, 6.4)	10.5	6.2 (5.0, 7.4)	33.9	23.7 (21.9, 25.5)
21:00h	6.1	7.3 (5.9, 8.7)	7.8	5.7 (4.7, 6.7)	12.7	11.5 (10.2, 12.7)
22:00h	3.3	6.8 (5.9, 7.8)	3.7	5.6 (4.6, 6.6)	3.9	6.1 (5.3, 7.0)
**Plant protein**‡						
05:00h	2.4	3.8 (3.5, 4.0)	3.1	3.8 (3.6, 4.0)	2.0	1.0 (0.7, 1.2)
06:00h	12.3	6.9 (6.3, 7.5)	8.3	5.6 (5.0, 6.3)	7.4	6.7 (5.9, 7.5)
07:00h	35.6	6.8 (6.4, 7.2)	28.8	6.6 (6.2, 7.1)	19.8	6.3 (5.8, 6.8)
08:00h	26.5	6.1 (5.7, 6.5)	29.6	6.7 (6.3, 7.1)	22.7	6.1 (5.7, 6.6)
09:00h	14.6	4.9 (4.4, 5.4)	17.8	5.5 (5.0, 6.0)	19.1	6.0 (5.5, 6.6)
10:00h	24.5	3.0 (2.7, 3.3)	25.3	3.7 (3.3, 4.0)	22.4	4.7 (4.3, 5.1)
11:00h	6.4	2.3 (1.8, 2.9)	18.6	2.5 (2.2, 2.9)	34.8	5.6 (5.2, 5.9)
12:00h	100.0	7.1 (6.9, 7.3)	1.4	13.3 (10.4, 16.2)	5.9	2.5 (1.8, 3.2)
13:00h	4.2	2.1 (1.5, 2.6)	98.0	7.1 (6.8, 7.3)	6.7	6.1 (5.1, 7.2)
14:00h	10.2	2.4 (2.0, 2.7)	6.7	2.1 (1.6, 2.6)	36.9	5.9 (5.5, 6.2)
15:00h	25.1	2.9 (2.6, 3.2)	20.9	2.8 (2.5, 3.1)	21.5	4.4 (4.0, 4.8)
16:00h	14.5	2.5 (2.2, 2.9)	16.1	3.1 (2.7, 3.5)	18.9	3.0 (2.6, 3.3)
17:00h	22.1	5.5 (5.0, 6.1)	15.1	4.1 (3.6, 4.7)	19.3	5.2 (4.6, 5.8)
18:00h	46.9	6.4 (6.0, 6.8)	39.5	7.4 (6.9, 7.9)	33.5	6.9 (6.3, 7.4)
19:00h	35.4	7.0 (6.5, 7.5)	40.8	7.1 (6.5, 7.6)	39.6	7.2 (6.6, 7.7)
20:00h	21.7	4.8 (4.3, 5.3)	23.9	5.3 (4.6, 5.9)	26.5	5.4 (4.8, 6.0)
21:00h	10.7	3.6 (3.1, 4.2)	12.2	3.4 (2.9, 3.9)	11.6	3.5 (3.0, 4.0)
22:00h	3.3	2.0 (1.6, 2.4)	4.7	2.1 (1.8, 2.4)	4.9	2.3 (1.9, 2.6)
23:00h	1.0	1.0 (0.9, 1.1)	0.5	0.2 (0.1, 0.3)	4.2	1.0 (1.0, 1.0)

*Class 1, 2, and 3 of total protein correspond to Class T1, T2, and T3, respectively.

†Class 1, 2, and 3 of animal protein correspond to Class A1, A2, and A3, respectively.

‡Class 1, 2, and 3 of plant protein correspond to Class P1, P2, and P3, respectively.
